# Supplementary material for: Docker4Circ: A Framework for the Reproducible Characterization of circRNAs from RNA-Seq Data
Source: Int J Mol Sci. 2019 Dec 31;21(1):293. doi: 10.3390/ijms21010293 (PMC6982331; doi:10.3390/ijms21010293)
Supplement: Supplementary file 1 [file ijms-21-00293-s001.zip › Supplementary_Materials.pdf]

Supplementary Material of "Docker4Circ: A framework for a reproducible characterization of circRNAs from RNA-Seq data"

Table of content

1. Docker4Circ installation

1.1 System requirements

1.2. *docker4seq* R package installation

1.3. Installation of the Graphical User Interface (GUI)

2. Docker4Circ Module 1: circRNAs prediction

2.1 CIRI2 circRNAs prediction

2.1.1. *bwaIndex* function: BWA reference definition

2.1.2. *bwa* function: BWA alignment

2.1.3. *ciri2* function: CIRI2 circRNAs prediction

2.1.4. *wrapperCiri* function: complete CIRI2 prediction from raw RNA-Seq reads

2.2 STARChip circRNA prediction

2.2.1. *rsemstarIndex* function: STAR reference definition

2.2.2. *starChimeric* function: detection of chimeric transcripts

2.2.3. *starChipIndex* function: reference definition for STARChip

2.2.4. *starchipCircle* function: STARChip circRNAs prediction

2.2.5. *wrapperSTARChip* function: complete STARChip prediction from raw RNA-Seq reads

2.3. Predictions overlap

2.3.1. *circrnaMergePredictions* function: merge and filter of circRNA predictions

2.3.1. *circrnaOverlapResults* function: overlap circRNA predictions

3. Docker4Circ Module 2: circRNAs annotation and classification

3.1. *circrnaPrepareFiles* function: exon and transcripts reference definition

3.2. *circrnaClassification* function: circRNAs classification

3.3. *circrnaAnnotations* function: circRNAs annotation using online databases

4. Docker4Circ Module 3: circRNAs sequence analysis

4.1. *circrnaBSJunctions* function: reconstruction of BS junction sequences

4.2. c*iri_as* function: detection of alternative internal circRNA structures

5. Docker4Circ Module 4: circRNAs expression analysis

5.1. *circrnaQuantification* function: quantification of circRNAs expression in RNA-Seq datasets

5.2. *mergeData* function: merge different output files

5.2. *wrapperDeseq2* function: differential expression analysis

6. Docker4Circ Graphical User Interface

7. References

1. Docker4Circ installation

The Docker4Circ functions are embedded in the *docker4seq* R package developed in the context of the Reproducible Bioinformatics Project (http://reproducible-bioinformatics.org/). Furthermore, the functions are implemented in the Java Graphical User Interface (GUI) 4SeqGUI (https://github.com/mbeccuti/4SeqGUI).

1.1 System requirements

The *docker4seq* R package requires:

- R 3.0.0 or higher (https://www.r-project.org/)
- Docker software. For more info see this document: https://docs.docker.com/engine/installation/

Docker4seq is expected to run on 64 bits linux machine with at least 4 cores.

The functions in *docker4seq* package require that user is sudo or part of a docker group. See the following document for more info: https://docs.docker.com/install/linux/linux-postinstall/

The first time *docker4seq* is installed the *downloadContainers* needs to be executed to download to the local repository the containers that are needed for the use of *docker4seq.*

The execution of the 4SeqGUI requires Java-Oracle SE Runtime Environment 8.0 or higher and R 3.0.0 or higher.

1.2. docker4seq R package installation

Docker4seq can be installed and executed using the following R code.

```
# Installation
install.packages("devtools")
library("devtools")
install_github("kendomaniac/docker4seq", ref="master")

# Program execution
library("docker4Seq")
```

1.3. Installation of the Graphical User Interface (GUI)

To install 4SeqGUI use the following code on a linux command line:

```
git clone https://github.com/mbeccuti/4SeqGUI.git
```

To execute 4SeqGUI please digit the following code:

```
cd /path_to_the_installation_folder/4SeqGUI
bash 4SeqGUI.sh
```

Each function in Docker4Circ and docker4seq function was implemented to take common parameters including:

- *group*, a character string. Two options: "sudo" or "docker", depending on which group the user belongs.
- *scratch_folder*, a character string indicating the scratch folder where docker container will be mounted

In the following sections, each function of the Docker4Circ pipeline is described.

2. Docker4Circ Module 1: circRNAs prediction

This module is designed to predict circRNAs starting from RNA-Seq reads in fastq format. The circRNA prediction algorithms that are currently implemented in this modules are CIRI2 (Gao et al., 2017) and STARChip (Akers et al., 2018).

2.1 CIRI2 circRNAs prediction

The circRNAs prediction using the CIRI2 algorithm requires a RNA-Seq reads alignment using BWA (Li et al., 2009). Then, an initial BWA indexing of the reference genome and the BWA read alignment is required. The BAM/SAM files generated from the alignment are then used as input for CIRI2.

2.1.1. *bwaIndex* function: BWA reference definition

This function executes the docker container *docker.io/repbioinfo/bwaindex* where BWA is installed. Based on a fasta file of a reference genome, the function returns the related BWA index that is required for the read alignment.

The R function can be used with the following syntax:

```
bwaIndex(group = c("sudo","docker"), scratch.folder, genome.folder,
genome.url, dbsnp.file, g1000.file, mode =
c("General","GATK","miRNA","ncRNA"), mb.version, mb.species, rc.version,
rc.species, length)
```

- *genome.folder*, a character string indicating the folder where the indexed reference genome for bwa will be located
- *genome.url*, a character string indicating the URL from download web page for the genome sequence of interest
- *dbsnp.file*, a character string indicating the name of dbSNP vcf located in the genome folder. The dbSNP vcf, dbsnp_138.b37.vcf.gz and dbsnp_138.hg19.vcf.idx.gz, can be downloaded from ftp://gsapubftp-anonymous@ftp.broadinstitute.org/bundle/b37
- *g1000.file*, a character string indicating the name of 1000 genome vcf located in the genome folder.
  The 1000 genomes vcf, Mills_and_1000G_gold_standard.indels.b37.vcf.gz and Mills_and_1000G_gold_standard.indels.hg19.sites.vcf.idx.gz, can be downloaded from ftp://gsapubftp-anonymous@ftp.broadinstitute.org/bundle/b37/
- *mode*, a character string indicating the required type of analysis. Compatible analyses mode are "General", "GATK", "miRNA", and "ncRNA". In "General" mode the url of any online fasta file ("genome.url" argument) can be provided and indexed. In the GATK analysis mode, the list of variants from dbsnp ("dbsnp.file" argument) and g1000 ("dbsnp.file" argument) are required in addition to the url of the genome fasta ("genome.url" argument). In "miRNA" analysis mode, the version ("mb.version" argument) and species prefix ("mb.species" argument) of miRBase (Kozomara et al.,

2018) are required. In "ncRNA" analysis mode, the version ("rc.version" argument) and species prefix ("rc.species" argument) of RNA Central (RNAcentral Consortium, 2016) are required. This mode require also a desidered maximum length of the studied RNA annotations ("length" argument).

- *rc.version*, a character string indicating the required version of RNA Central database. Visit ftp://ftp.ebi.ac.uk/pub/databases/RNAcentral/releases/ to select the proper version id.
- *rc.species*, a character string indicating the name of a species annotated in RNA Central (e.g. "Homo sapiens" for human ncRNAs). Please refer to NCBI taxonomy annotations at https://www.ncbi.nlm.nih.gov/Taxonomy/Browser/wwwtax.cgi to proper species name.
- *length*, an integer corresponding on the length threshold selected to define the ncRNA reference from RNA Central.

For circRNA analysis, this function can be used directly from the command line using the following syntax:

```
docker run -v genome.folder:/data/scratch -d docker.io/repbioinfo/bwaindex
bash /bin/bwa.index.sh genome.folder General genome.url
```

The arguments *genome.folder* and *genome.url* correspond to those used for the R function.

2.1.2. *bwa* function: BWA alignment

This function executes the docker container *docker.io/repbioinfo/bwa.2019.01* where BWA is installed. Based on the input fastq files and the indexed reference genome, the function returns:

- the file dedup_reads.bam, the sorted and duplicates marked BAM file
- dedup_reads.bai, the index of the dedup_reads.bam
- dedup_reads.stats, which provides mapping statistics

The R function can be used with the following syntax:

```
bwa(group = c("sudo","docker"), fastq.folder, scratch.folder,
genome.folder, seq.type = c("se","pe"), threads = 1, sample.id)
```

- *fastq.folder*, a character string indicating where gzip fastq files are located
- *genome.folder*, a character string indicating the folder where the indexed reference genome for bwa is located
- *seq.type*, a character string indicating the type of reads to be trimmed. Two options: "se" or "pe" respectively for single end and pair end sequencing
- *threads*, a number indicating the number of cores to be used from the application
- *sample.id*, a character string indicating the unique id to be associated to the bam that will be created

The function can be used directly from the command line using the following syntax:

```
docker run -v scratch.folder:/data/scratch genome.folder:/data/genome -v
fastq.folder:/fastq.folder -d docker.io/repbioinfo/bwa.2019.01 bash
/bin/bwa_pe.sh threads fastq_R1 fastq_R2 /data/genome sample.id
genome.folder
```

The arguments *scratch.folder, genome.folder, fastq.folder, threads,* and *sample.id* correspond to those used for the R function. The arguments *fastq_R1* and *fastq_R2* correspond to the names of the two paired fastq files reporting the sequencing reads.

2.1.3. *ciri2* function: CIRI2 circRNAs prediction

This function executes the docker container *docker.io/repbioinfo/docker4circ.2019.01* where CIRI v2.0.6 is installed and it provides the list of circRNAs predicted from a RNA-Seq experiment. For CIRI 2 tool details refer to (Gao et al., 2017).

The output of this function is a list of CIRI2 predicted circRNAs.
The R function can be used with the following syntax:

```
ciri2(group = c("sudo", "docker"), sam.file, scratch.folder, genome.file,
annotation.file, max.span = 200000, stringency.value = c("high", "low",
"zero"), quality.threshold = 10, threads = 1)
```

- *sam.file*, a character string indicating the path to the RNA-Seq alignment SAM file from BWA
- *genome.file,* a character string indicating the path to the fasta file of the reference genomic sequence (it should be the same reference indexed used for the BWA alignment)
- *annotation.file*, a character string indicating the path to the GTF/GFF file reporting the reference gene annotations
- *max.span*, an integer reporting the maximum spanning distance of a circRNA (default = 200,000 bp)
- *stringency.value*, the selected stringency level of the analysis. Three possible options are available: "high" (high stringency, default), in which CIRI2 only provides circRNAs supported by more than 2 distinct Paired Chiastic Clipping (PCC) signals; "low" (low stringency), CIRI2 only provides circRNAs supported by more than 2 junction reads; "zero", CIRI2 provides all circRNAs regardless junction read counts or PCC signals
- *quality.threshold*, integer indicating the threshold for mapping quality of each segment of junction reads (default = 10)
- *threads*, integer indicating the number of threads used for the analysis (default = 1)

The function can be used directly from the command line using the following syntax:

```
docker run -v scratch.folder:/data/scratch -v sam.file:/data/samfile -v
genome.file:/data/reference -v data.folder:/data/out -v
annotation.file/:/data/annotation.gtf/gff -d
docker.io/repbioinfo/docker4circ.2019.01 python3 /ciri2/docker4ciri.py
ciri2 --stringency stringency -S max.span -T threads -U quality.threshold
--anno
```

The arguments *scratch.folder, genome.file, sam.file, annotation.file, threads, stringency.value, max.span,* and *sample.id* correspond to those used for the R function. The argument *data.folder* correspond to the folder in which the output files will be stored.

2.1.4. *wrapperCiri* function: complete CIRI2 prediction for raw RNA-Seq reads

This function calls sequentially the docker containers for the FASTQC analysis, the BWA alignment, and CIRI2 circRNA prediction starting from the raw RNA-Seq reads.

The R function can be used with the following syntax:

```
wrapperCiri(group = c("sudo", "docker"), scratch.folder, data.folder,
genome.file, seq.type = c("se", "pe"), sample.id, threads = 1,
annotation.file, max.span = 200000, stringency.value = c("high", "low",
"zero"), quality.threshold = 10)
```

- *data.folder*, a character string indicating where gzip fastq files are located
- *genome.file*, a character string indicating the path to the Fasta file of the reference genomic sequence (it should be the same reference indexed for the BWA alignment)
- *seq.type*, a character string indicating the type of reads to be trimmed. Two options: "se" or "pe" respectively for single end and pair end sequencing
- *sample.id*, a character string indicating the unique id to be associated to the bam that will be created
- *threads*, a number indicating the number of cores to be used from the application
- sam.file, a character string indicating the path to the RNA-Seq alignment SAM file from BWA
- *annotation.file*, a character string indicating the path to the GTF/GFF file reporting the reference gene annotations
- max.span, an integer reporting the maximum spanning distance of a circRNA (default = 200,000 bp)
- *stringency.value*, the selected stringency level of the analysis. Three possible options are available: "high" (high stringency, default), in which CIRI2 only provides circRNAs supported by more than 2 distinct PCC signals; "low" (low stringency), CIRI2 only provides circRNAs supported by more than 2 junction reads; "zero", CIRI2 provides all circRNAs regardless junction read counts or PCC signals
- *quality.threshold*, integer indicating the threshold for mapping quality of each segment of junction reads (default=10)

2.2. STARChip circRNAs prediction

The circRNAs prediction using the STARChip algorithm requires a RNA-Seq reads alignment using STAR Chimeric (Akers et al., 2018). Then, the initial indexing of the reference genome using STAR followed by the STAR chimeric read alignment is required. The output files generated from the alignment are then used as input for STARChip.

2.2.1. *rsemstarIndex* function: STAR reference definition

This function executes the docker container docker.io/repbioinfo/rsemstar.2017.01 where RSEM and STAR are installed. The index is created using ENSEMBL genome fasta file. User needs to provide the URL for ENSEMBL genome and annotations located in the ENSEMBL ftp.

The R function can be used with the following syntax:

```
rsemstarIndex(group = c("sudo","docker"),  genome.folder=,
ensembl.urlgenome, ensembl.urlgtf, threads = 1)
```

- *genome.folder*, a character string indicating the folder where the indexed reference genome for bwa will be located
- *ensembl.urlgenome*, a character string indicating the URL from ENSEMBL ftp for the unmasked genome sequence of interest
- *ensembl.urlgtf*, a character string indicating the URL from ENSEMBL ftp for the GTF for genome of interest
- *threads*, a number indicating the number of cores to be used from the application

The function can be used directly from the command line using the following syntax:

```
docker run -v scratch.folder:/data/scratch -v data.folder:/data/ -d
docker.io/repbioinfo/rsemstar.2019.02 bash /bin/rsemstar.index.sh
genome.folder ensembl.urlgenome ensembl.urlgtf threads
```

The arguments *scratch.folder, data.folder, genome.folder, ensembl.urlgenome, ensembl.urlgtf,* and *threads* correspond to those used for the R function.

2.2.2. *starChimeric* function: detection of chimeric transcripts

The function executes the docker *docker.io/repbioinfo/star251.2017.01* running STAR chimeric to detect chimeric transcripts on paired-end sequences.

The R function can be used with the following syntax:

```
starChimeric(group = c("sudo","docker"), fastq.folder = getwd(),
scratch.folder, genome.folder, threads = 1, chimSegmentMin = 20,
chimJunctionOverhangMin = 15)
```

- *fastq.folder*, a character string indicating where gzip fastq files are located
- *genome.folder*, a character string indicating the folder where the indexed reference genome for STAR is located.
- *threads*, a number indicating the number of cores to be used from the application
- *chimSegmentMin*, is a positive integer indicating the minimal length of the overlap of a read to the chimeric element
- *chimJunctionOverhangMin*, is a positive integer indicating the minimum overhang for a chimeric junction

The function can be used directly from the command line using the following syntax:

```
docker run -v scratch.folder:/data/scratch -v fastq.folder:/fastq.folder -v
genome.folder:/genome -d docker.io/repbioinfo/star251.2019.02 /bin/bash
/bin/start_chimeric.sh chimSegmentMin chimJunctionOverhangMin threads
fastq_R1 fastq_R2
```

The arguments *scratch.folder, fastq.folder, genome.folder, chimSegmentMin chimJunctionOverhangMin,* and *threads* correspond to those used for the R function. The arguments *fastq_R1* and *fastq_R2* correspond to the names of the two paired fastq files reporting the sequencing reads.

2.2.3. *starChipIndex* function: reference definition for STARChip

The function executes the Docker *docker.io/repbioinfo/star251.2017.01* to prepare the bed file required by STARChip to detect circular RNAs on paired-end sequences. The genome folder and the reference annotation GTF file must be the same used by starChimeric.

The R function can be used with the following syntax:

```
starChipIndex(group = c("sudo","docker"), genome.folder)
```

- *genome.folder*, a character string indicating the folder where the indexed reference genome for STAR is located.

The function can be used directly from the command line using the following syntax:

```
docker run -v scratch.folder:/data/scratch -v genome.folder:/genome -d
docker.io/repbioinfo/star251.2017.01 sh /bin/starchipIndex.sh
```

The arguments *scratch.folder, fastq.folder* and *genome.folder c*orrespond to those used for the R function.

2.2.4. *starchipCircle* function: STARChip circRNAs prediction

This function runs the Docker *docker.io/repbioinfo/star251.2017.01* in which STARChip algorithm is installed to perform a circRNAs detection from paired-end RNA-Seq data. The function considers the folders containing the output of *starChimeric* function. It also requires a specific bed generated with *starChipIndex* function in the genome folder used by *starChimeric*.

The R function can be used with the following syntax:

```
starchipCircle(group = c("sudo","docker"), genome.folder, scratch.folder,
samples.folder, reads.cutoff, min.subject.limit, threads, do.splice =
c("True", "False"), cpm.cutoff=0, subjectCPM.cutoff = 0, annotation =
c("true", "false"))
```

- *genome.folder*, a character string indicating the folder where the indexed reference genome for STAR is located.
- *reads.cutoff*, Integer. Minimum number of back-splicing reads required
- *min.subject.limit*, Integer. Minimum number of individuals with readsCutoff reads required to carry forward a circRNA for analysis
- *threads*, Integer. Number of threads to use
- *do.splice*, true false. The splices within the circRNA be detected and reported. Linear splices are searched within each cRNA in each individual. Any linear splice with >= 60\% of the read count of the circRNA is considered a splice within the circRNA. Two files are then created, "*.consensus"* with most common splice pattern, and "*.allvariants"* with all reported splice patterns.
- *cpm.cutoff*, Float. Reads counts are loaded into R and log2(CountsPerMillion) is calculated using the limma package. With cpmCutoff > 0, circRNA with log2(CPM) below this value will be filtered from this analysis
- *subjectCPM.cutoff*, Integer. See above. This value is the lower limit for number of individuals required to have the circRNA expressed at a value higher than cpmCutoff.
- *annotation*, true/false. circRNA are provided with gene annotations
- *samples.folder*, the folder where are located all the folders of the samples processed with starChimeric

The function can be used directly from the command line using the following syntax:

```
docker run -v scratch.folder:/data/scratch -v samples.folder:/samples -v
genome.folder:/genome -d docker.io/repbioinfo/star251.2017.02 bin/bash
/bin/start_starchipCircle.sh reads.cutoff min.subject.limit threads
do.splice cpm.cutoff subjectCPM.cutoff annotation
```

The arguments *scratch.folder, sample.folder, genome.folder, reads.cutoff, min.subject.limit, threads, do.splice, cpm.cutoff* and *subjectCPM.cutoff annotation* correspond to those used for the R function.

2.2.5. *wrapperSTARChip* function: complete STARChip prediction for raw RNA-Seq reads
This function calls sequentially the Docker containers for FASTQC, STAR, and STARChip to predict the list of circRNAs starting from the raw RNA-Seq reads.

The R function can be used with the following syntax:

```
wrapperSTARChip(group = c("sudo","docker"), genome.folder, scratch.folder,
samples.folder, reads.cutoff, min.subject.limit, threads, do.splice =
c("True", "False"), cpm.cutoff=0, subjectCPM.cutoff = 0, annotation =
c("true", "false"), chimSegmentMin = 20, chimJunctionOverhangMin = 15)
```

- *genome.folder*, a character string indicating the folder where the indexed reference genome for STAR is located.
- *samples.folder,* the folder where are located all the subfolders of the samples processed with starChimeric.
- *threads*, Integer. Number of threads to use
- *chimSegmentMin*, is a positive integer indicating the minimal length of the overlap of a read to the chimeric element
- *chimJunctionOverhangMin*, is a positive integer indicating the minimum overhang for a chimeric junction
- *reads.cutoff*, Integer. Minimum number of back-splicing reads required
- *min.subject.limit*, Integer. Minimum number of individuals with readsCutoff reads required to carry forward a circRNA for analysis
- *do.splice*, true false. The splices within the circRNA be detected and reported. Linear splices are searched within each cRNA in each individual. Any linear splice with >= 60\% of the read count of the circRNA is considered a splice within the circRNA. Two files are then created, "*.consensus"* with most common splice pattern, and "*.allvariants"* with all reported splice patterns.
- *cpm.cutoff*, Float. Reads counts are loaded into R and log2(CountsPerMillion) is calculated using the limma package. With cpmCutoff > 0, circRNA with log2(CPM) below this value will be filtered from this analysis
- *subjectCPM.cutoff*, Integer. See above. This value is the lower limit for number of individuals required to have the circRNA expressed at a value higher than cpmCutoff.
- *annotation*, true/false. circRNA are provided with gene annotations.

2.3.1. *circrnaMergePredictions* function: merge and filter of CIRI2 predictions

This function executes the docker container *docker.io/cursecatcher/ciri2* by performing the merging of circRNAs lists detected by different tools following the user indications. Prediction made using the same prediction tool are merged based on the suffix of the file (for example all CIRI2 prediction must be named with the suffix .ciri2). Support tools are CFS (suffix *cfs*), CIRI (*ciri*), CIRI2 (*ciri2*), Find_Circ2 (*findcirc2*), CIRCexplorer (*circexplorer*), CIRCexplorer2 (*circexplorer2*), DCC (*dcc*), KNIFE (*knife*), STARChip (*starchip*), Uroborus (*uroborus*), and circRNA_Finder (*circrnafinder*). The function executes also a filter based on the number of reads computed in each experiment and across replicates of the same biological condition.

The R function can be used with the following syntax:

```
circrnaMergePrediction(group = c("sudo", "docker"), scratch.folder,
data.folder, samples.list, covariates.list, covariate.order, min_reads = 2,
min_reps = 0, min_avg = 10, used.tool = c("ciri2", "circexplorer2"))
```

- *data.folder*, a character string indicating the data folder where output files are located
- *samples.list,* a character vector indicating the identifiers of the samples
- *covariates.list*, a character vector indicating the classes of the samples
- *covariate.order*, a character vector indicating the order of covariates in the output files
- *min_reads*, the minimum number of reads supporting a circRNA and detected in at least min_reps number of replicates of the same experimental condition (default = 2)
- *min_reps,* the minimum number of replicates with at least min_reads (default = 0)
- *min_avg*, the average number of back-splicing reads across biological replicates of the same experimental condition that shall support a circRNA (default = 10)
- used.tool, a character string indicating the tool used for the prediction

The function can be used directly from the command line using the following syntax:

```
docker run -v scratch.folder:/data/scratch -v data.folder:/data/ -d
docker.io/repbioinfo/docker4circ.2019.02 python3 /ciri2/docker4ciri.py
merge --sample samples.list --cov covariates.list --order covariate.order
--mr min_reads --mrep min_reps --avg min_avg --tool used.tool
```

The arguments *scratch.folder, data.folder, sample.list covariates.list, covariate.order, min_reads, min_reps, min_avg* correspond to those used for the R function. The output of the function are two tables. The first table contains the BS read counts detected for each circRNA in each experiment. The second table provides the circRNAs information in TAB separated columns. Every row of the file reports information of a single circRNA indicating:

• the chromosome name specified as chr## (*i.e.* chr1)
• the circRNA start position (*i.e* the 5' genomic position)
• the end position (*i.e* the 3' genomic position)
• the circRNA name specified as chr##_start_end (*i.e.* chr1_10_100)
• the genomic strand specified as 1 (forward) or -1 (reverse)

2.3.2. ***circrnaOverlapResults*** **function: Overlap circRNA predictions**

This function executes the docker container docker4circ to overlap circRNA predictions obtained from multiple tools. The function automatically searches within a user-defined folder, each circRNAs prediction tables named with the suffix “tool.txt” where *tool* is the name of the tool used for the prediction. Then, the function provides the list of circRNAs with indication of the number and the name of the tools which predict them. Support tools are ACFS (suffix *cfs*), CIRI (*ciri*), CIRI2 (*ciri2*), Find_Circ2 (*findcirc2*), CIRCexplorer (*circexplorer*), CIRCexplorer2 (*circexplorer2*), DCC (*dcc*), KNIFE (*knife*), STARChip (*starchip*), Uroborus (*uroborus*), and circRNA_Finder (*circrnafinder*).

The R function can be used with the following syntax:

```
circrnaOverlapResults(group = c("sudo", "docker"), scratch.folder,
input.folder, output.folder, min_support)
```

- *input.folder*, a string indicating the path of the folder containing the predictions to overlap
- *output.folder*, string indicating the path of the output folder
- *version*, used.aligner, a string indicating the aligner used to generate the junctions.file.
- min_support, an integer representing the minimum number of occurrences that a circRNA has to be in order to be reported

The function can be used directly from the command line using the following syntax:

The function can be used directly from the command line using the following syntax:

```
docker run -v scratch.folder:/data/scratch -v input.folder:/data/input -v
output.folder:/data/out -d docker.io/repbioinfo/docker4circ.2019.02 python3
/ciri2/docker4ciri.py overlap -t min_support
```

3. Docker4Circ Module 2: circRNAs annotation and classification

3.1. *circrnaPrepareFiles* function: exon and transcripts reference definition

This function executes the Docker container *docker.io/repbioinfo/docker4circ.2019.01* by running the circRNAs classification module of CircHunter. For CircHunter algorithm details refer to Coscujuela Tarrero et al., 2018.

The R function can be used with the following syntax:

```
circrnaPrepareFiles(group = c("sudo","docker"), scratch.folder,
data.folder, assembly = c("hg18", "hg38", "hg19", "mm9", "mm10", "rn6",
"dm6", "ce11"), version=NULL)
```

- *data.folder*, a string indicating the data folder where the output files will be saved
- *assembly*, string indicating the reference human genome assembly. Compatible assemblies: hg19 (default), hg18, hg38, mm9, mm10, rn6, dm6, ce11.
- *version*, Ensembl database version used for the analysis. If no version number is provided, the last version is considered.

The function generates two files reporting respectively the exons and the transcript isoform annotations based on the ENSEMBL annotations. The exon file reports the information of each exon of a desidered genome assembly and each row contains the following data:

Field	Description	Example
Gene ID	Ensembl Gene ID	ENSG01
Transcript ID	Ensembl Transcript ID	ENST01
Exon ID	Ensembl Exon ID	ENSE03
Chromosome	Chromosome name	chr1
Exon start	Start position of the exon	70
Exon end	End position of the exon	90
Strand	Exon strand	1
Rank	Rank of the exon	3
Gene start	Start position of the gene	1
Gene end	End position of the gene	125
Transcript start	Start position of the transcript	10
Transcript end	End position of the transcript	120

The Ensembl isoform data file is used by Docker4Circ to obtain a unique circRNAs classification and it reports the following data:

Field	Description	Example
Gene ID	Ensembl Gene ID	ENSG01
Transcript ID	Ensembl Transcript ID	ENST02
Chromosome	Chromosome name	chr1
Transcript name	Isoform identifier	Isoform-201

The function can be used directly from the command line using the following syntax:

```
docker run -v scratch.folder:/data/scratch -v data.folder:/data -d
docker.io/repbioinfo/docker4circ.2019.01 Rscript
/scripts/circhunter/circhunter.R --preparedata -as assembly -of -v version
```

The arguments *scratch.folder, data.folder,* and *assembly* correspond to those used for the R function.

3.2. ***circrnaClassification*** **function: circRNAs classification based on Ensembl annotations**

This function executes the Docker container *docker.io/repbioinfo/docker4circ.2019.01* by running the circRNAs CircHunter classification analysis of a set of circRNAs. For CircHunter algorithm details refer to (Coscujuela Tarrero et al., 2018).

The function will output a transcript-wide classification of the provided circRNAs (circRNA_classification file) where every row represents a transcript associated with a circRNA and consequently the classification of that circRNA referring to the transcript. The function also provides a unique circRNAs classification in which every circRNA is assigned to a unique classification.

The R function can be used with the following syntax:

```
circrnaClassification(group = c("sudo","docker"), scratch.folder,
circrna.data, exon.data, isoform.data, assembly = c("hg18",  "hg19",
"hg38", "mm9", "mm10", "rn6", "dm6", "ce11"), version=NULL)
```

- *circrna.data*, string indicating the path to the list of circRNAs
- *exon.data*, string indicating the path to the exon annotation file
- *isoform.data*, string indicating the path to the isoform annotation file
- *assembly*, string indicating the reference human genome assembly. Compatible assemblies: hg19 (default), hg18, hg38, mm9, mm10, rn6, dm6, ce11.

The function can be used directly from the command line using the following syntax:

```
docker run -v scratch.folder:/data/scratch -v data.folder:/data/out -v
isoform.data:/data/isoformdata -v exon.data:/data/genome -v
circrna.data:/data/circRNA -d docker.io/repbioinfo/docker4circ.2019.01
Rscript /scripts/circhunter/circhunter.R --classification -as assembly -sg
-id -of -v version
```

The arguments *scratch.folder, exone.data, isoform.data,* and *assembly c*orrespond to those used for the R function. Conversely *data.folder* is the folder storing the circRNA set.

3.3. *circrnaAnnotations* function: circRNAs annotation using online databases

This function executes the Docker container *docker.io/repbioinfo/docker4circ.2019.01* in the annotation mode to overlap a list of circRNAs with the annotations from circBase (Glažar et al., 2017), TSCD (Xia et al., 2016), CSCD v2 (Xia et al., 2017), ExoRBase (Li et al., 2017), Circ2Disease (Yao et al., 2018), CircFunBase (Meng et al., 2019). The function also converts the circRNAs genomic coordinates between human genome assembly using the *pyliftover* Python package. This conversion is performed whether the assembly used for circRNAs prediction (that must be indicated as argument) is different from the assembly used by the compared database.

The R function can be used with the following syntax:

```
circrnaAnnotations(group = c("sudo", "docker"), scratch.folder, ciri.file,
annotation.sources=c("circbase", "tscd", "cscd", "exorbase","circ2disease",
"circfunbase"), assembly=c("hg18", "hg19", "hg38", "mm9", "mm10"))
```

- *ciri.file*, a list of circRNAs derived from a CIRI2 prediction analysis
- *assembly*, a character string indicating the reference genome assembly. The function currently work with the hg18, hg19 and hg38 human genome assemblies
- *Annotation.sources*, a vector of character strings indicating the circRNA databases to analyse

The function can be used directly from the command line using the following syntax:

```
docker run -v scratch.folder:/data/scratch -v data.folder:/data/out -v
ciri.file:/data/cirifile -d docker.io/repbioinfo/docker4circ.2019.02
python3 /ciri2/docker4ciri.py annotation -s annotation.sources -v assembly
```

The arguments *scratch.folder, annotation.sources* and *assembly* correspond to those used for the R function. Conversely *data.folder* is the folder storing the circRNA set.

4. Docker4Circ Module 3: circRNAs sequence analysis

This module of Docker4Circ is designed to analyse and reconstruct the sequence of a set of circRNAs.

4.1. *circrnaBSJunctions* function: reconstruction of BS junction sequences

This function executes the Docker container *docker.io/repbioinfo/docker4circ.2019.01* by running the circRNA back-splicing sequence reconstruction module of CircHunter starting from a set of circRNAs. For CircHunter algorithm details please refer to (Coscujuela Tarrero et al., 2018).

Sequences are outputted in a FASTA format file named *circRNA_backsplicing_sequences.fasta.*

The R function can be used with the following syntax:

```
circrnaBSJunctions(group = c("sudo", "docker"), scratch.folder,
circrna.data, exon.data, assembly = c("hg18", "hg19", "hg38", "mm9",
"mm10", "dm6", "rn6", "ce11")
```

- *circrna.data*, string indicating the path to the list of circRNAs
- *exon.data*, string indicating the path to the exon annotation file
- *assembly*, string indicating the reference human genome assembly. Compatible assemblies: hg19 (default), hg18, hg38

The function can be used directly from the command line using the following syntax:

```
docker run -v scratch.folder:/data/scratch -v data.folder:/data/out -v
circrna.data:/data/circRNA -v exon.data:/data/genome -d
docker.io/repbioinfo/docker4circ.2019.01 Rscript
/scripts/circhunter/circhunter.R --sequences -as assembly -of -sg
```

The arguments *scratch.folder, circrna.data, exon.data* and *assembly c*orrespond to those used for the R function. Conversely *data.folder* is the folder storing the circRNA set.

4.2. c*iri_as* function: detection of alternative internal circRNA structures

This function executes the Docker container *docker.io/repbioinfo/docker4circ.2019.01* where CIRI-AS algorithm (Gao et al., 2016) is installed. The function returns the list of alternative internal structures of a set of input circRNAs.

The R function can be used with the following syntax:

```
ciri_as(group = c("sudo", "docker"), scratch.folder, sam.file, ciri.file,
genome.file, annotation.file)
```

- *sam.file*, a character string indicating the path to the RNA-Seq alignment SAM/BAM file from BWA
- *ciri.file*, a character string indicating the path to the list of circRNAs
- *genome.file*, a character string indicating the path to the Fasta file of the reference genomic sequence (it should be the same reference indexed for the BWA alignment)
- *annotation.file*, a character string indicating the path to the GTF/GFF file reporting the reference gene annotations
-

The function can be used directly from the command line using the following syntax:

```
docker run -v scratch.folder:/data/scratch -v sam.file:/data/samfile -v
ciri.file:/data/cirifile -v genome.file:/data/reference -v
data.folder:/data/out -v annotation.file:/data/annotation.GTF/GFF -d
docker.io/repbioinfo/docker4circ.2019.01 python3 /ciri2/docker4ciri.py
structure --anno
```

The arguments *scratch.folder, sam.file, ciri.file, genome.file* and *annotation.file c*orrespond to those used for the R function. Conversely *data.folder* is the folder storing the circRNA set.

5. Docker4Circ Module 4: circRNAs expression analysis

This module of Docker4Circ is designed to perform several analysis about the circRNAs expression using different RNA-Seq experiments.

5.1. *circrnaQuantification* function: quantification of circRNAs expression in RNA-Seq datasets

This function executes the docker container *docker.io/repbioinfo/docker4circ.2019.01* by running the circRNAs quantification module of CircHunter to quantify the level of expression of a set of circRNA BS sequences in a given RNA-Seq experiment. For CircHunter algorithm details please refer to: https://github.com/carlo-deintinis/circhunter/tree/master/CircHunter and (Coscujuela Tarrero et al., 2018).

The R function can be used with the following syntax:

```
circrnaQuantification(group = c("sudo", "docker"), scratch.folder,
rnaseq.data, backsplicing_junctions.data, hc.params)
```

- *rnaseq.data*, string indicating the path to the fastq file of the RNA-Seq dataset to analyse
- *backsplicing_junctions.data*, string indicating the path to the fasta file of the circRNA back-splicing sequences to search in the RNA-Seq dataset
- *hc.params*, vector of six parameters to set the analysis. The element of the vector indicate in order:
    - the k-mer size
    - the thread number
    - the dimension of the hash table
    - the dimension of the collision list
    - the number of k-mers that must be matched to the sequence to consider the sequence itself as represented in the RNA-Seq data
    - the number of perfect matches to consider the k-mer matched to a sequence

The function can be used directly from the command line using the following syntax:

```
docker run -v scratch.folder:/data/scratch -v data.folder:/data/out -v
rnaseq.data:/data/rnaseq -v backsplicing_junctions.data:/data/bksj -d
docker.io/repbioinfo/docker4circ.2019.01 Rscript
/scripts/circhunter/circhunter.R --readcount -of -hc hc.params --samplename
samplename
```

The arguments *scratch.folder, rnaseq.data, hc.params,* and *backsplicing_junctions.data* correspond to those used for the R function. Conversely *data.folder* is the folder storing the circRNA set and samplename is the name of the dataset analysed.

5.2. ***mergeData*** function: merge different output data

This function runs the Docker container *docker.io/repbioinfo/docker4circ.2019.01* which executes the *merge_data* function to merge different files characterized by the same extension. Given a folder and a vector of sample identifiers, the function will iterate over each folder with a name belonging to these identifiers. A covariate vector can be also provided to group each sample into a specific group.

The R function can be used with the following syntax:

```
mergeData(group = c("sudo","docker"), data.folder=getwd(), samples.ids,
covariates, covariate.order, extension, column_index)
```

- *data.folder*, a character string indicating the data folder where the file to merge are located
- *samples.ids,* a character vector indicating the identifiers of the samples
- *covariates,* a character vector indicating the classes of the samples
- *covariate.order*, a character vector indicating a vector reporting the covariate classes ordered as desidered in the output file
- *extension*, a character string indicating the filename extension of the files that have to merge
- *column_index*, an integer value > 1 indicating which column values have to be reported in the output file

The function can be used directly from the command line using the following syntax:

```
docker run -v scratch.folder:/data/scratch -v data.folder:/data/out -d
docker.io/repbioinfo/docker4circ.2019.01 merge_data --sample samples.ids
--cov covariates --order covariate.order --col column_index --ext extension
```

The arguments *scratch.folder, data.folder, samples.ids, covariates, covariate.order, column_index,* and *extension* correspond to those used for the R function.

5.2. ***wrapperDeseq2*** **function: differential expression analysis**

This function runs the Docker container *docker.io/repbioinfo/r332.2017.01* which executes the *DESeq2* R function (Love et al., 2014) on a table generated with *sample2experiment* or *mergeData* function having the covariates added in the names of the columns, separated by the names with underscores. The function returns a full table of differentially expressed genes (prefix DEfull), a filtered table of differentially expressed genes (prefix DEfiltered) and the normalized counts table (prefix normalized)

The R function can be used with the following syntax:

```
wrapperDeseq2(output.folder, group = c("sudo","docker"), experiment.table,
log2fc = 1, fdr = 0.1, ref.covar = "0", type = c("gene", "isoform",
"mirna"), batch = FALSE)
```

- *output.folder*, a character string indicating where the tables generated by samples2experiment or *mergeData* are located and were results will be placed
- *experiment.table*, a character string indicating the counts table generated with sample2experiment or *mergeData* with addition of covariates
- *log2fc*, log2fc threshold for differentially expressed genes
- *fdr*, fdr threshold
- *ref.covar*, covariate to be used as reference
- *type*, character with three options: gene, isoform, mirna. if gene is used two files are generated for gene set enrichment, the filtered Gene symbols and the background that contains all gene symbols.
- *batch*, logical FALSE, TRUE

The function can be used directly from the command line using the following syntax:

```
docker run -v scratch.folder:/data/scratch -v output.folder:/data/scratch
-d docker.io/repbioinfo/r332.2017.01 Rscript /bin/.wrapperDeseq2.R
experiment.table log2fc fdr ref.covar type batch
```

The arguments *scratch.folder, output.folder, experiment.table, log2fc, fdr, ref.covar, type* and *batch* correspond to those used for the R function.

6. Docker4Circ Graphical User Interface

Docker4Circ can be executed using the GUI 4SeqGUI to easily set an analysis process. Each module of Docker4Circ can be applied, including circRNAs classification (Supplementary Figure 1), circRNAs classification and annotation analysis, circRNAs sequence analysis (Supplementary Figure 2), and circRNAs expression analysis (Supplementary Figure 3).

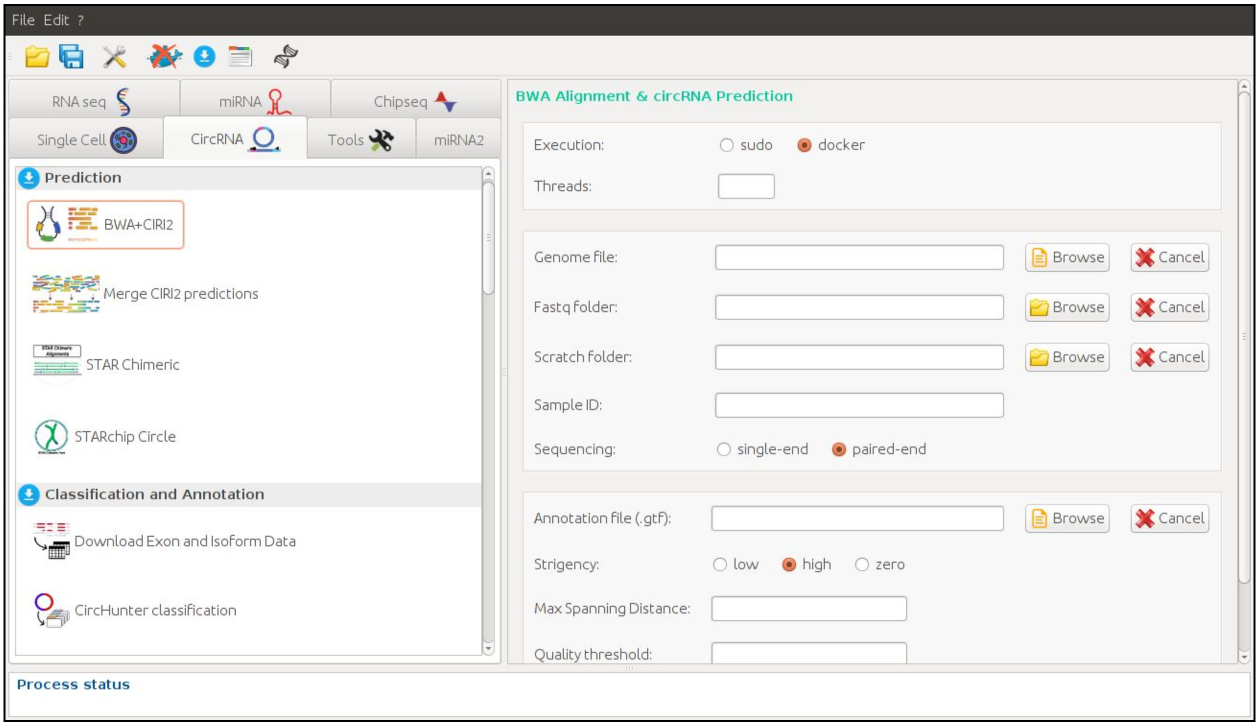

Supplementary Figure 1. Image from the 4SeqGUI Graphical User Interface (GUI) at the CircRNA section (collecting the Docker4Circ functions) with a detail on the circRNAs prediction module.

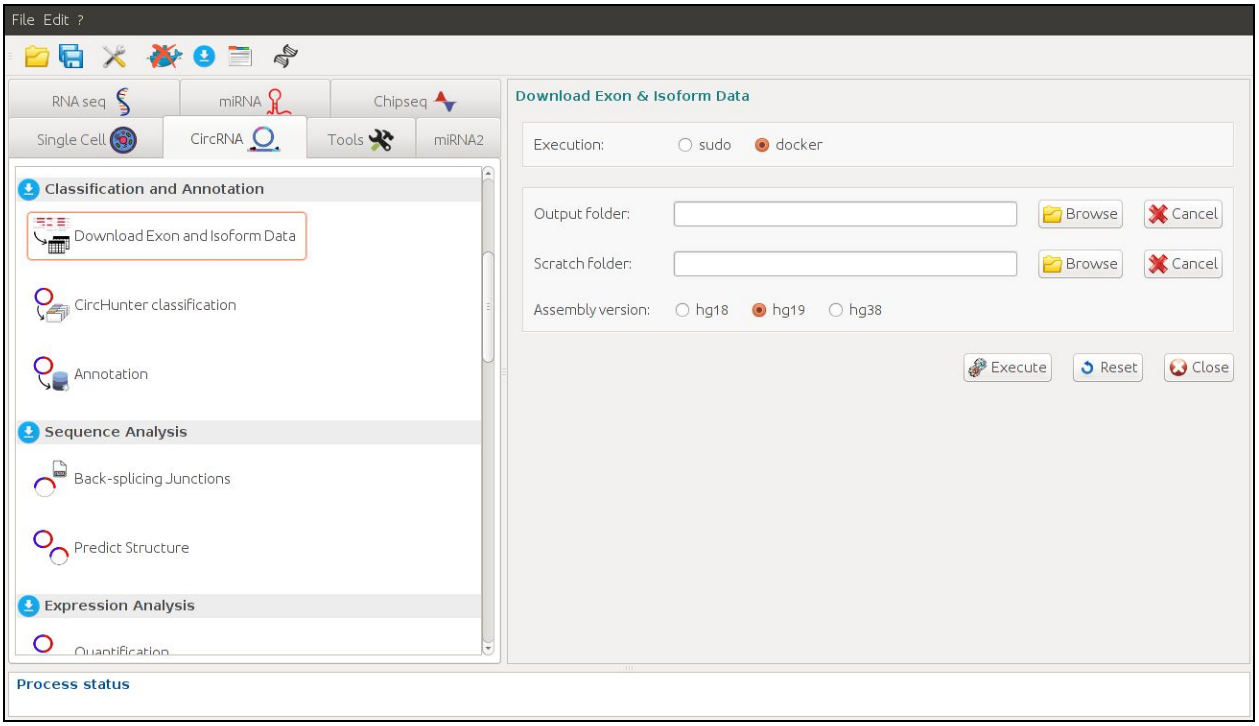

Supplementary Figure 2. Image from the 4SeqGUI Graphical User Interface (GUI) at the CircRNA section (collecting the Docker4Circ functions) with a detail on the circRNA annotation/classification and sequence analysis modules.

7. References

Akers, N. K., Schadt, E. E., Losic, B., & Valencia, A. (2018). STAR Chimeric Post for rapid detection of circular RNA and fusion transcripts. *Bioinformatics*, 34(14):2364-2370.

Coscujuela Tarrero, L., Ferrero, G., Miano, V., De Intinis, C., Ricci, L., Arigoni, M., et al. (2018). Luminal breast cancer-specific circular RNAs uncovered by a novel tool for data analysis. *Oncotarget*, 9(18), 14580.

Gao, Y., Wang, J., Zheng, Y., Zhang, J., Chen, S., & Zhao, F. (2016). Comprehensive identification of internal structure and alternative splicing events in circular RNAs. *Nature communications*, 7, 12060.

Gao, Y., Zhang, J., & Zhao, F. (2017). Circular RNA identification based on multiple seed matching. *Briefings in bioinformatics*, 19(5):803-810.

Glažar, P., Papavasileiou, P., & Rajewsky, N. (2014). circBase: a database for circular RNAs. *RNAs*, 20(11), 1666-1670.

Kozomara, A., Birgaoanu, M., & Griffiths-Jones, S. (2018). miRBase: from microRNA sequences to function. *Nucleic acids research*, 47(D1):D155-D162.

Li, H., & Durbin, R. (2009). Fast and accurate short read alignment with Burrows–Wheeler transform. *Bioinformatics*, 25(14), 1754-1760.

Li, S., Li, Y., Chen, B., Zhao, J., Yu, S., Tang, Y., et al. (2017). exoRBase: a database of circRNA, lncRNA and mRNA in human blood exosomes. *Nucleic acids research*, 46(D1), D106-D112.

Love, M. I., Huber, W., & Anders, S. (2014). Moderated estimation of fold change and dispersion for RNA-seq data with DESeq2. *Genome Biology*, 15(12), 550.

Meng, X., Hu, D., Zhang, P., Chen, Q., & Chen, M. (2019). CircFunBase: a database for functional circular RNAs. *Database*, 2019.

RNAcentral Consortium. (2016). RNAcentral: a hub of information for non-coding RNA sequences. *Nucleic acids research*. 47(D1):D1250-D1251.

Xia, S., Feng, J., Lei, L., Hu, J., Xia, L., Wang, J., et al. (2016). Comprehensive characterization of tissue-specific circular RNAs in the human and mouse genomes. *Briefings in bioinformatics*, 18(6), 984-992.

Xia, S., Feng, J., Chen, K., Ma, Y., Gong, J., Cai, F., et al. (2017). CSCD: a database for cancer-specific circular RNAs. *Nucleic acids research*, 46(D1), D925-D929.

Yao, D., Zhang, L., Zheng, M., Sun, X., Lu, Y., & Liu, P. (2018). Circ2Disease: a manually curated database of experimentally validated circRNAs in human disease. *Scientific reports*, 8(1), 11018.
